# Supplementary material for: Identification of highly-protective combinations of Plasmodium vivax recombinant proteins for vaccine development
Source: eLife. 2017 Sep 26;6:e28673. doi: 10.7554/eLife.28673 (PMC5655538; doi:10.7554/eLife.28673)
Supplement: Figure 2—source data 1. — Geom mean = geometric mean. Exposure was defined as the total number of P. vivax blood-stage clones acquired per time-at-risk (molFOB) and lifetime exposure as age multiplied by molFOB. Geometric mean IgG levels and 95% intervals are in arbitrary units interpolated form standard curves using a 5PL logistic regression model and were multiplied by 1000. For age and lifetime exposure, rho (r) and P values were calculated using Spearman's rank test. For infection status, IgG levels were log10 transformed and P values calculated using unpaired 2-tailed t tests. P values < 0.05 were deemed significant. [file elife-28673-fig2-data1.docx]

**Figure 2 – source data 1:** **Associations between IgG to *P. vivax* antigens with measures of concurrent and cumulative exposure in Papua New Guinean children aged 1-3 years.** Geom mean = geometric mean. Exposure was defined as the total number of P. vivax blood-stage clones acquired per time-at-risk (molFOB) and lifetime exposure as age multiplied by molFOB. Geometric mean IgG levels and 95% intervals are in arbitrary units interpolated form standard curves using a 5PL logistic regression model and were multiplied by 1000. For age and lifetime exposure, rho (r) and P values were calculated using Spearman's rank test. For infection status, IgG levels were log10 transformed and P values calculated using unpaired 2-tailed t tests. P values <0.05 were deemed significant.

|  | Age | | | | | |  | Lifetime exposure | | | | | |  | Infection status | | | | | | |
| --- | --- | --- | --- | --- | --- | --- | --- | --- | --- | --- | --- | --- | --- | --- | --- | --- | --- | --- | --- | --- | --- |
|  | **All children** | | **PCR -** | | **PCR +** | |  | **All children** | | **PCR +** | | **PCR -** | |  | **PCR -** | | | **PCR +** | | |  |
| **Antigen** | rho | P value | rho | P value | rho | P value |  | rho | P value | rho | P value | rho | P value |  | Geom mean | 95%CI | | Geom mean | 95%CI | | P  value |
| **PVX_081550** | 0.26 | **<0.001** | 0.33 | **0.001** | 0.19 | **0.037** |  | 0.30 | **<0.001** | 0.41 | **<0.001** | 0.08 | 0.390 |  | 0.022 | 0.018 | 0.028 | 0.039 | 0.033 | 0.048 | **<0.001** |
| **ARP** | 0.15 | **0.023** | 0.17 | 0.093 | 0.14 | 0.122 |  | 0.07 | 0.282 | 0.06 | 0.593 | 0.09 | 0.310 |  | 0.386 | 0.343 | 0.436 | 0.412 | 0.368 | 0.462 | 0.446 |
| **GAMA** | 0.11 | 0.106 | 0.16 | 0.114 | 0.07 | 0.435 |  | 0.11 | 0.109 | 0.15 | 0.156 | 0.06 | 0.522 |  | 0.006 | 0.005 | 0.007 | 0.007 | 0.006 | 0.010 | 0.168 |
| **P41** | -0.07 | 0.311 | 0.02 | 0.831 | -0.17 | 0.057 |  | -0.04 | 0.538 | 0.03 | 0.797 | -0.20 | **0.022** |  | 0.013 | 0.010 | 0.017 | 0.026 | 0.019 | 0.034 | **0.001** |
| **P12** | 0.02 | 0.765 | 0.04 | 0.693 | -0.03 | 0.774 |  | 0.00 | 0.987 | -0.03 | 0.775 | -0.04 | 0.649 |  | 0.012 | 0.009 | 0.016 | 0.031 | 0.022 | 0.042 | **<0.001** |
| **CyRPA** | 0.28 | **<0.001** | 0.27 | **0.006** | 0.26 | **0.003** |  | 0.36 | **<0.001** | 0.40 | **<0.001** | 0.20 | **0.026** |  | 0.273 | 0.192 | 0.387 | 0.919 | 0.656 | 1.289 | **<0.001** |
| **CSP** | 0.03 | 0.646 | -0.01 | 0.957 | 0.06 | 0.535 |  | 0.11 | 0.118 | 0.10 | 0.344 | 0.07 | 0.467 |  | 0.126 | 0.097 | 0.165 | 0.166 | 0.132 | 0.210 | 0.124 |
| **MSP9 N-term** | 0.24 | **<0.001** | 0.23 | **0.024** | 0.23 | **0.011** |  | 0.27 | **<0.001** | 0.27 | **0.008** | 0.20 | **0.028** |  | 0.079 | 0.062 | 0.103 | 0.105 | 0.086 | 0.129 | 0.089 |
| **DBPII Sal1** | 0.06 | 0.378 | 0.04 | 0.715 | 0.05 | 0.549 |  | 0.11 | 0.109 | 0.12 | 0.260 | 0.04 | 0.685 |  | 0.191 | 0.157 | 0.231 | 0.281 | 0.230 | 0.344 | **0.007** |
| **DBPII P** | 0.16 | **0.015** | 0.23 | **0.019** | 0.09 | 0.343 |  | 0.26 | **<0.001** | 0.34 | **<0.001** | 0.14 | 0.135 |  | 0.173 | 0.133 | 0.224 | 0.296 | 0.233 | 0.375 | **0.003** |
| **DBPII O** | 0.14 | **0.031** | 0.16 | 0.108 | 0.11 | 0.220 |  | 0.23 | **<0.001** | 0.27 | **0.008** | 0.14 | 0.123 |  | 0.252 | 0.198 | 0.322 | 0.422 | 0.340 | 0.525 | **0.002** |
| **DBPII C** | 0.15 | **0.022** | 0.22 | **0.032** | 0.09 | 0.341 |  | 0.25 | **<0.001** | 0.33 | **0.001** | 0.13 | 0.139 |  | 0.164 | 0.126 | 0.213 | 0.296 | 0.233 | 0.374 | **0.001** |
| **DBPII AH** | 0.07 | 0.309 | 0.05 | 0.653 | 0.08 | 0.387 |  | 0.16 | **0.020** | 0.20 | **0.048** | 0.05 | 0.561 |  | 0.187 | 0.154 | 0.227 | 0.290 | 0.240 | 0.350 | **0.002** |
| **MSP3a full** | 0.12 | 0.073 | 0.12 | 0.244 | 0.12 | 0.174 |  | 0.22 | **0.001** | 0.26 | **0.012** | 0.11 | 0.222 |  | 1.028 | 0.873 | 1.210 | 1.307 | 1.130 | 1.511 | **0.031** |
| **MSP3a**  **block 1** | 0.17 | **0.010** | 0.15 | 0.149 | 0.17 | 0.055 |  | 0.31 | **<0.001** | 0.33 | **0.001** | 0.22 | **0.016** |  | 0.685 | 0.592 | 0.792 | 0.881 | 0.788 | 0.984 | **0.006** |
| **MSP3a**  **block 2** | 0.07 | 0.285 | 0.00 | 0.962 | 0.13 | 0.158 |  | 0.22 | **0.001** | 0.19 | 0.071 | 0.18 | **0.045** |  | 0.510 | 0.427 | 0.608 | 0.568 | 0.492 | 0.655 | 0.346 |
| **MSP3a**  **C-term** | 0.17 | **0.011** | 0.16 | 0.105 | 0.16 | 0.084 |  | 0.22 | **0.001** | 0.17 | 0.110 | 0.20 | **0.028** |  | 0.088 | 0.071 | 0.109 | 0.132 | 0.112 | 0.155 | **0.003** |
| **MSP3a**  **N-term** | 0.07 | 0.287 | 0.04 | 0.659 | 0.11 | 0.236 |  | 0.21 | **0.002** | 0.18 | 0.087 | 0.19 | **0.040** |  | 1.183 | 0.992 | 1.411 | 1.357 | 1.177 | 1.566 | 0.226 |
| **MSP1 19** | -0.12 | 0.080 | -0.08 | 0.439 | -0.15 | 0.094 |  | -0.11 | 0.114 | -0.08 | 0.431 | -0.17 | 0.060 |  | 0.376 | 0.279 | 0.508 | 0.557 | 0.423 | 0.734 | 0.058 |
| **AMA1** | -0.07 | 0.289 | -0.11 | 0.255 | -0.03 | 0.731 |  | 0.06 | 0.359 | 0.03 | 0.783 | 0.03 | 0.765 |  | 0.378 | 0.306 | 0.466 | 0.440 | 0.372 | 0.519 | 0.258 |
| **RBP1a** | 0.07 | 0.301 | 0.11 | 0.292 | 0.00 | 0.970 |  | 0.19 | **0.005** | 0.21 | **0.046** | 0.06 | 0.532 |  | 0.308 | 0.247 | 0.385 | 0.512 | 0.422 | 0.621 | **0.001** |
| **RBP2a** | -0.02 | 0.802 | 0.01 | 0.950 | -0.04 | 0.623 |  | 0.11 | 0.099 | 0.20 | 0.055 | -0.05 | 0.595 |  | 0.543 | 0.421 | 0.700 | 1.259 | 0.974 | 1.628 | **<0.001** |
| **RBP2b** | 0.21 | **0.002** | 0.18 | 0.069 | 0.22 | **0.012** |  | 0.31 | **<0.001** | 0.31 | **0.002** | 0.22 | **0.013** |  | 0.797 | 0.629 | 1.011 | 1.639 | 1.380 | 1.946 | **<0.001** |
| **RBP2cNB** | 0.01 | 0.825 | -0.06 | 0.550 | 0.08 | 0.385 |  | 0.09 | 0.187 | 0.07 | 0.514 | 0.06 | 0.541 |  | 0.339 | 0.269 | 0.427 | 0.457 | 0.363 | 0.575 | 0.074 |
| **RBP2-P2** | 0.08 | 0.240 | 0.01 | 0.903 | 0.13 | 0.158 |  | 0.21 | **0.002** | 0.20 | 0.056 | 0.14 | 0.126 |  | 1.349 | 1.117 | 1.631 | 2.013 | 1.715 | 2.364 | **0.002** |
| **PVX_094350** | 0.25 | **<0.001** | 0.32 | **0.001** | 0.17 | 0.061 |  | 0.27 | **<0.001** | 0.29 | **0.004** | 0.17 | 0.056 |  | 1.221 | 1.047 | 1.424 | 1.634 | 1.437 | 1.858 | **0.004** |
| **AKLP** | 0.24 | **<0.001** | 0.20 | **0.044** | 0.26 | **0.003** |  | 0.34 | **<0.001** | 0.30 | **0.003** | 0.29 | **0.001** |  | 1.074 | 0.897 | 1.285 | 1.623 | 1.389 | 1.897 | **0.001** |
| **PVX_087670** | 0.17 | **0.012** | 0.19 | 0.065 | 0.14 | 0.130 |  | 0.25 | **<0.001** | 0.24 | **0.020** | 0.15 | 0.102 |  | 1.367 | 1.170 | 1.598 | 2.036 | 1.782 | 2.326 | **<0.001** |
| **RhopH2** | 0.15 | **0.023** | 0.11 | 0.290 | 0.17 | 0.053 |  | 0.24 | **<0.001** | 0.21 | **0.046** | 0.20 | **0.022** |  | 1.164 | 0.981 | 1.381 | 1.632 | 1.424 | 1.872 | **0.002** |
| **PVX_122805** | 0.16 | **0.017** | 0.11 | 0.297 | 0.19 | **0.035** |  | 0.26 | **<0.001** | 0.21 | **0.040** | 0.20 | **0.023** |  | 1.730 | 1.487 | 2.012 | 2.319 | 2.054 | 2.618 | **0.003** |
| **CCp5** | 0.28 | **<0.001** | 0.31 | **0.002** | 0.26 | **0.004** |  | 0.33 | **<0.001** | 0.35 | **0.001** | 0.24 | **0.007** |  | 1.433 | 1.226 | 1.676 | 1.924 | 1.666 | 2.222 | **0.007** |
| **PVX_114330** | 0.16 | **0.016** | 0.09 | 0.353 | 0.21 | **0.020** |  | 0.26 | **<0.001** | 0.18 | 0.074 | 0.25 | **0.006** |  | 1.821 | 1.582 | 2.096 | 2.483 | 2.217 | 2.783 | **0.001** |
| **Pv-fam-a/**  **PVX_088820** | 0.15 | **0.025** | 0.10 | 0.329 | 0.17 | 0.061 |  | 0.24 | **<0.001** | 0.22 | **0.033** | 0.16 | 0.078 |  | 2.001 | 1.744 | 2.295 | 2.728 | 2.435 | 3.056 | **0.001** |
| **Pv-fam-a/**  **PVX_092995** | 0.06 | 0.352 | 0.03 | 0.742 | 0.08 | 0.397 |  | 0.19 | **0.005** | 0.15 | 0.139 | 0.13 | 0.156 |  | 1.589 | 1.382 | 1.827 | 2.090 | 1.877 | 2.327 | **0.002** |
| **PVX_080665** | 0.19 | **0.004** | 0.14 | 0.172 | 0.24 | **0.006** |  | 0.27 | **<0.001** | 0.18 | 0.082 | 0.30 | **0.001** |  | 0.595 | 0.498 | 0.711 | 0.756 | 0.658 | 0.868 | **0.034** |
| **RAMA** | 0.01 | 0.909 | -0.01 | 0.946 | 0.00 | 0.997 |  | 0.17 | **0.013** | 0.11 | 0.288 | 0.14 | 0.130 |  | 1.231 | 1.047 | 1.447 | 1.641 | 1.425 | 1.891 | **0.009** |
| **SERA** | 0.20 | **0.002** | 0.19 | 0.052 | 0.22 | **0.015** |  | 0.28 | **<0.001** | 0.27 | **0.009** | 0.24 | **0.006** |  | 0.805 | 0.690 | 0.938 | 1.112 | 0.964 | 1.282 | **0.003** |
| **EBP** | 0.26 | **<0.001** | 0.26 | **0.009** | 0.26 | **0.003** |  | 0.38 | **<0.001** | 0.43 | **<0.001** | 0.26 | **0.003** |  | 0.265 | 0.201 | 0.350 | 0.547 | 0.424 | 0.706 | **<0.001** |
