# Supplementary material for: Identification of highly-protective combinations of Plasmodium vivax recombinant proteins for vaccine development
Source: eLife. 2017 Sep 26;6:e28673. doi: 10.7554/eLife.28673 (PMC5655538; doi:10.7554/eLife.28673)
Supplement: Figure 3—source data 1. — M = medium IgG levels; H = high IgG levels; uIRR = Unadjusted incidence rate ratio; aIRR = Adjusted incidence rate ratio, adjusted for exposure (molFOB), age, village of residency, and season. P values were from GEE models and were deemed significant if <0.05. [file elife-28673-fig3-data1.docx]

**Figure 3 – source data 1: Associations between antibodies to 38 *P. vivax* proteins and risk of *P. vivax* clinical episodes (> 500 parasites/μL) in Papua New Guinean children aged 1-3 years.** M = medium IgG levels; H = high IgG levels; uIRR = Unadjusted incidence rate ratio; aIRR = Adjusted incidence rate ratio, adjusted for exposure (molFOB), age, village of residency, and season. P values were from GEE models and were deemed significant if <0.05.

| **Antigen** | **uIRR** | **95%CI** | | **P value** | **aIRR** | **95%CI** | | **P value** |
| --- | --- | --- | --- | --- | --- | --- | --- | --- |
| PVX_081550 M | 0.76 | 0.54 | 1.05 | 0.10 | **0.74** | **0.55** | **0.99** | **0.041** |
| PVX_081550 H | **0.41** | **0.29** | **0.60** | **<0.001** | **0.46** | **0.33** | **0.64** | **<0.001** |
| ARP M | 0.93 | 0.66 | 1.32 | 0.68 | 0.98 | 0.73 | 1.32 | 0.91 |
| ARP H | 1.00 | 0.69 | 1.46 | 0.98 | 0.88 | 0.63 | 1.23 | 0.47 |
| GAMA M | 1.12 | 0.80 | 1.57 | 0.51 | 1.03 | 0.75 | 1.40 | 0.87 |
| GAMA H | 0.82 | 0.55 | 1.23 | 0.34 | 0.75 | 0.54 | 1.04 | 0.08 |
| P41 M | 0.96 | 0.68 | 1.36 | 0.83 | 0.89 | 0.67 | 1.18 | 0.41 |
| P41 H | **0.63** | **0.43** | **0.93** | **0.019** | **0.56** | **0.41** | **0.77** | **<0.001** |
| P12 M | 1.05 | 0.75 | 1.47 | 0.79 | 0.96 | 0.71 | 1.29 | 0.77 |
| P12 H | 0.69 | 0.47 | 1.02 | 0.06 | **0.65** | **0.47** | **0.91** | **0.012** |
| CyRPA M | **0.57** | **0.41** | **0.79** | **0.001** | **0.55** | **0.41** | **0.73** | **<0.001** |
| CyRPA H | **0.40** | **0.28** | **0.57** | **<0.001** | **0.47** | **0.34** | **0.66** | **<0.001** |
| CSP M | 1.04 | 0.71 | 1.43 | 0.82 | 0.90 | 0.67 | 1.21 | 0.50 |
| CSP H | 0.90 | 0.61 | 1.30 | 0.59 | 0.82 | 0.59 | 1.13 | 0.22 |
| MSP9 N-term M | 0.78 | 0.55 | 1.10 | 0.16 | 0.87 | 0.64 | 1.18 | 0.37 |
| MSP9 N-term H | **0.54** | **0.37** | **0.78** | **0.001** | **0.64** | **0.45** | **0.90** | **0.011** |
| DBPII Sal1 M | 1.02 | 0.70 | 1.47 | 0.93 | 0.88 | 0.63 | 1.22 | 0.5 |
| DBPII Sal1 H | 0.88 | 0.61 | 1.27 | 0.49 | **0.70** | **0.51** | **0.96** | **0.026** |
| DBPII P M | 0.75 | 0.52 | 1.08 | 0.12 | 0.72 | 0.51 | 1.02 | 0.06 |
| DBPII P H | **0.65** | **0.46** | **0.93** | **0.017** | **0.61** | **0.44** | **0.83** | **0.002** |
| DBPII O M | 0.76 | 0.53 | 1.10 | 0.15 | 0.76 | 0.55 | 1.05 | 0.10 |
| DBPII O H | **0.66** | **0.46** | **0.95** | **0.026** | **0.64** | **0.45** | **0.90** | **0.010** |
| DBPII C M | 0.73 | 0.51 | 1.06 | 0.10 | 0.72 | 0.51 | 1.01 | 0.06 |
| DBPII C H | **0.65** | **0.46** | **0.93** | **0.019** | **0.64** | **0.47** | **0.89** | **0.007** |
| DBPII AH M | 0.87 | 0.60 | 1.26 | 0.46 | 0.74 | 0.54 | 1.02 | 0.07 |
| DBPII AH H | 0.76 | 0.53 | 1.08 | 0.13 | **0.63** | **0.46** | **0.86** | **0.004** |
| MSP3a full M | 0.91 | 0.64 | 1.27 | 0.57 | 0.90 | 0.66 | 1.22 | 0.49 |
| MSP3a full H | **0.52** | **0.35** | **0.76** | **0.001** | **0.51** | **0.36** | **0.73** | **<0.001** |
| MSP3a block 1 M | 0.98 | 0.69 | 1.38 | 0.904 | 0.99 | 0.72 | 1.36 | 0.94 |
| MSP3a block 1 H | **0.55** | **0.38** | **0.80** | **0.002** | **0.63** | **0.44** | **0.91** | **0.013** |
| MSP3a block 2 M | 0.76 | 0.54 | 1.08 | 0.13 | 0.86 | 0.62 | 1.20 | 0.37 |
| MSP3a block 2 H | **0.56** | **0.39** | **0.80** | **0.002** | **0.56** | **0.41** | **0.76** | **<0.001** |
| MSP3a C-term M | **0.66** | **0.47** | **0.92** | **0.014** | 0.83 | 0.61 | 1.12 | 0.22 |
| MSP3a C-term H | **0.56** | **0.38** | **0.83** | **0.004** | **0.62** | **0.44** | **0.87** | **0.005** |
| MSP3a N-term M | 0.90 | 0.63 | 1.28 | 0.55 | 0.90 | 0.67 | 1.23 | 0.52 |
| MSP3a N-term H | **0.56** | **0.39** | **0.81** | **0.002** | **0.56** | **0.40** | **0.79** | **0.001** |
| MSP1 19 M | 0.89 | 0.61 | 1.31 | 0.56 | 0.87 | 0.64 | 1.20 | 0.41 |
| MSP1 19 H | 1.31 | 0.91 | 1.89 | 0.14 | 0.93 | 0.66 | 1.30 | 0.68 |
| AMA1 M | 0.75 | 0.52 | 1.08 | 0.13 | **0.65** | **0.47** | **0.90** | **0.008** |
| AMA1 H | 0.88 | 0.61 | 1.28 | 0.50 | **0.66** | **0.48** | **0.90** | **0.010** |
| RBP1a M | 0.77 | 0.56 | 1.07 | 0.12 | 0.79 | 0.59 | 1.06 | 0.11 |
| RBP1a H | **0.53** | **0.36** | **0.78** | **0.001** | **0.56** | **0.40** | **0.76** | **<0.001** |
| RBP2a M | 0.87 | 0.60 | 1.25 | 0.45 | 0.77 | 0.57 | 1.03 | 0.08 |
| RBP2a H | 0.82 | 0.57 | 1.18 | 0.30 | **0.69** | **0.50** | **0.94** | **0.018** |
| RBP2b M | **0.52** | **0.38** | **0.73** | **<0.001** | **0.63** | **0.47** | **0.84** | **0.002** |
| RBP2b H | **0.49** | **0.34** | **0.71** | **<0.001** | **0.54** | **0.38** | **0.77** | **0.001** |
| RBP2cNB M | 0.84 | 0.59 | 1.21 | 0.36 | 0.89 | 0.65 | 1.21 | 0.45 |
| RBP2cNB H | **0.68** | **0.48** | **0.96** | **0.031** | **0.67** | **0.49** | **0.91** | **0.010** |
| RBP2-P2 M | **0.64** | **0.45** | **0.91** | **0.014** | **0.68** | **0.50** | **0.94** | **0.019** |
| RBP2-P2 H | **0.66** | **0.46** | **0.94** | **0.020** | **0.63** | **0.46** | **0.86** | **0.004** |
| PVX_094350 M | 0.97 | 0.69 | 1.35 | 0.84 | 0.89 | 0.65 | 1.23 | 0.48 |
| PVX_094350 H | **0.55** | **0.37** | **0.81** | **0.003** | **0.64** | **0.45** | **0.91** | **0.014** |
| AKLP2 M | 0.71 | 0.50 | 1.02 | 0.06 | 0.79 | 0.57 | 1.11 | 0.17 |
| AKLP2 H | **0.57** | **0.40** | **0.81** | **0.002** | **0.66** | **0.48** | **0.91** | **0.010** |
| PVX_087670 M | 0.93 | 0.66 | 1.29 | 0.65 | 0.92 | 0.68 | 1.24 | 0.57 |
| PVX_087670 H | **0.61** | **0.41** | **0.90** | **0.013** | **0.68** | **0.48** | **0.97** | **0.035** |
| RhopH2 M | 0.74 | 0.53 | 1.04 | 0.08 | 0.77 | 0.57 | 1.05 | 0.10 |
| RhopH2 H | **0.56** | **0.38** | **0.83** | **0.004** | **0.61** | **0.43** | **0.86** | **0.005** |
| PVX_122805 M | 0.89 | 0.64 | 1.24 | 0.49 | 0.90 | 0.67 | 1.21 | 0.49 |
| PVX_122805 H | **0.52** | **0.35** | **0.76** | **0.001** | **0.58** | **0.42** | **0.81** | **0.001** |
| CCp5 M | 0.82 | 0.59 | 1.15 | 0.25 | 0.87 | 0.64 | 1.19 | 0.38 |
| CCp5 H | **0.56** | **0.39** | **0.83** | **0.003** | **0.67** | **0.47** | **0.94** | **0.019** |
| PVX_114330 M | 0.88 | 0.62 | 1.23 | 0.44 | 0.90 | 0.67 | 1.21 | 0.50 |
| PVX_114330 H | **0.64** | **0.43** | **0.94** | **0.023** | **0.68** | **0.48** | **0.97** | **0.033** |
| Pv-fam-a/PVX_088820 M | 0.81 | 0.58 | 1.14 | 0.23 | 0.83 | 0.61 | 1.13 | 0.24 |
| Pv-fam-a/PVX_088820 H | **0.57** | **0.39** | **0.83** | **0.003** | **0.63** | **0.45** | **0.88** | **0.007** |
| Pv-fam-a/PVX_092995 M | 0.73 | 0.51 | 1.04 | 0.08 | 0.78 | 0.57 | 1.06 | 0.11 |
| Pv-fam-a/PVX_092995 H | 0.75 | 0.52 | 1.08 | 0.12 | **0.73** | **0.54** | **1.01** | **0.05** |
| PVX_080665 M | **0.68** | **0.48** | **0.98** | **0.036** | **0.71** | **0.51** | **0.99** | **0.041** |
| PVX_080665 H | **0.70** | **0.49** | **1.00** | **0.047** | **0.73** | **0.53** | **1.00** | **0.05** |
| RAMA M | 0.71 | 0.49 | 1.03 | 0.07 | **0.68** | **0.49** | **0.94** | **0.019** |
| RAMA H | 0.99 | 0.70 | 1.38 | 0.94 | 0.77 | 0.57 | 1.03 | 0.08 |
| SERA M | 0.83 | 0.59 | 1.17 | 0.29 | 0.80 | 0.60 | 1.07 | 0.14 |
| SERA H | **0.64** | **0.44** | **0.93** | **0.020** | **0.63** | **0.44** | **0.89** | **0.008** |
| EBP M | **0.64** | **0.47** | **0.89** | **0.007** | **0.68** | **0.51** | **0.90** | **0.007** |
| EBP H | **0.40** | **0.28** | **0.57** | **<0.001** | **0.44** | **0.31** | **0.63** | **<0.001** |
