# Supplementary material for: Identification of highly-protective combinations of Plasmodium vivax recombinant proteins for vaccine development
Source: eLife. 2017 Sep 26;6:e28673. doi: 10.7554/eLife.28673 (PMC5655538; doi:10.7554/eLife.28673)
Supplement: Figure 3—source data 2. — M = medium IgG levels; H = high IgG levels; uIRR = Unadjusted incidence rate ratio; aIRR = Adjusted incidence rate ratio; adjusted for exposure (molFOB), age, village of residency and season. P values were from GEE models and were deemed significant if <0.05. [file elife-28673-fig3-data2.docx]

**Figure 3 – source data 2:** **Associations between antibodies to 38 *P. vivax* proteins and risk of *P. falciparum* clinical episodes (> 2,500 parasites/μL) in Papua New Guinean children aged 1-3 years.** M = medium IgG levels; H = high IgG levels; uIRR = Unadjusted incidence rate ratio; aIRR = Adjusted incidence rate ratio; adjusted for exposure (molFOB), age, village of residency and season. P values were from GEE models and were deemed significant if <0.05.

| **Antigen** | **uIRR** | **95%CI** | | **P value** | **aIRR** | **95%CI** | | ***P* value** |
| --- | --- | --- | --- | --- | --- | --- | --- | --- |
| PVX_081550 M | **1.29** | **1.00** | **1.65** | **0.047** | 1.16 | 0.95 | 1.43 | 0.15 |
| PVX_081550 H | **1.43** | **1.12** | **1.83** | **0.004** | 0.92 | 0.75 | 1.14 | 0.46 |
| ARP M | 0.95 | 0.75 | 1.21 | 0.69 | 0.99 | 0.83 | 1.19 | 0.95 |
| ARP H | 0.81 | 0.65 | 1.02 | 0.07 | 0.88 | 0.72 | 1.08 | 0.23 |
| GAMA M | **0.75** | **0.58** | **0.96** | **0.022** | 0.89 | 0.72 | 1.10 | 0.28 |
| GAMA H | 1.01 | 0.81 | 1.26 | 0.93 | 0.89 | 0.75 | 1.06 | 0.18 |
| P41 M | 0.98 | 0.77 | 1.24 | 0.87 | 0.97 | 0.79 | 1.19 | 0.76 |
| P41 H | 0.95 | 0.76 | 1.20 | 0.69 | 1.09 | 0.90 | 1.32 | 0.38 |
| P12 M | 0.91 | 0.72 | 1.15 | 0.45 | 1.10 | 0.90 | 1.34 | 0.35 |
| P12 H | 0.97 | 0.76 | 1.24 | 0.81 | 1.18 | 0.97 | 1.44 | 0.10 |
| CyRPA M | **1.63** | **1.28** | **2.08** | **<0.001** | **1.25** | **1.01** | **1.55** | **0.040** |
| CyRPA H | **1.57** | **1.24** | **1.99** | **<0.001** | 1.06 | 0.85 | 1.32 | 0.62 |
| CSP M | 1.16 | 0.92 | 1.46 | 0.22 | 1.01 | 0.83 | 1.23 | 0.94 |
| CSP H | 1.05 | 0.83 | 1.32 | 0.67 | 0.91 | 0.75 | 1.11 | 0.37 |
| MSP9 N-term M | **1.41** | **1.09** | **1.83** | **0.009** | 1.16 | 0.94 | 1.43 | 0.15 |
| MSP9 N-term H | **1.60** | **1.25** | **2.04** | **<0.001** | 1.18 | 0.96 | 1.46 | 0.12 |
| DBPII P M | **1.61** | **1.24** | **2.08** | **<0.001** | **1.24** | **1.00** | **1.53** | **0.045** |
| DBPII P H | **1.89** | **1.46** | **2.45** | **<0.001** | **1.31** | **1.05** | **1.64** | **0.018** |
| DBPII O M | **1.54** | **1.19** | **1.98** | **0.001** | **1.26** | **1.03** | **1.55** | **0.026** |
| DBPII O H | **1.71** | **1.33** | **2.21** | **<0.001** | 1.21 | 0.97 | 1.52 | 0.093 |
| DBPII C M | **1.69** | **1.30** | **2.19** | **<0.001** | **1.33** | **1.07** | **1.65** | **0.009** |
| DBPII C H | **1.91** | **1.47** | **2.47** | **<0.001** | **1.30** | **1.04** | **1.64** | **0.023** |
| DBPII AH M | 1.22 | 0.95 | 1.56 | 0.13 | 1.07 | 0.87 | 1.31 | 0.54 |
| DBPII AH H | 1.23 | 0.98 | 1.56 | 0.08 | 1.09 | 0.90 | 1.33 | 0.37 |
| DBPII Sal1 M | 1.19 | 0.93 | 1.53 | 0.17 | 1.09 | 0.88 | 1.35 | 0.42 |
| DBPII Sal1 H | **1.32** | **1.04** | **1.67** | **0.022** | 1.17 | 0.97 | 1.42 | 0.11 |
| MSP3a C-term M | **1.45** | **1.12** | **1.87** | **0.004** | 1.11 | 0.90 | 1.38 | 0.32 |
| MSP3a C-term H | **1.64** | **1.29** | **2.08** | **<0.001** | 1.09 | 0.88 | 1.35 | 0.42 |
| MSP3a full M | **1.44** | **1.13** | **1.83** | **0.003** | 1.05 | 0.85 | 1.30 | 0.67 |
| MSP3a full H | **1.41** | **1.12** | **1.77** | **0.004** | 1.05 | 0.86 | 1.29 | 0.61 |
| MSP3a block 1 M | **1.34** | **1.03** | **1.75** | **0.027** | 1.06 | 0.85 | 1.31 | 0.61 |
| MSP3a block 1 H | **1.67** | **1.30** | **2.14** | **<0.001** | 1.08 | 0.88 | 1.33 | 0.47 |
| MSP3a block 2 M | **1.30** | **1.02** | **1.67** | **0.037** | 1.00 | 0.81 | 1.25 | 0.97 |
| MSP3a block 2 H | **1.44** | **1.12** | **1.85** | **0.004** | 1.06 | 0.86 | 1.30 | 0.57 |
| MSP3a N-term M | **1.45** | **1.13** | **1.85** | **0.003** | 1.08 | 0.87 | 1.33 | 0.49 |
| MSP3a N-term H | **1.44** | **1.13** | **1.84** | **0.003** | 1.04 | 0.84 | 1.29 | 0.70 |
| MSP1 19 M | 1.06 | 0.85 | 1.32 | 0.60 | 1.03 | 0.84 | 1.26 | 0.79 |
| MSP1 19 H | **0.74** | **0.57** | **0.94** | **0.015** | 1.02 | 0.83 | 1.25 | 0.86 |
| AMA1 M | **1.30** | **1.03** | **1.65** | **0.029** | 1.04 | 0.85 | 1.27 | 0.71 |
| AMA1 H | 1.06 | 0.83 | 1.36 | 0.65 | 1.06 | 0.86 | 1.30 | 0.57 |
| RBP1a | 1.19 | 0.93 | 1.54 | 0.17 | 0.91 | 0.74 | 1.12 | 0.38 |
| RBP1a | **1.31** | **1.04** | **1.66** | **0.024** | 1.00 | 0.83 | 1.20 | 1.00 |
| RBP2a | 0.97 | 0.75 | 1.25 | 0.81 | 1.10 | 0.89 | 1.36 | 0.38 |
| RBP2a | 1.13 | 0.89 | 1.42 | 0.33 | 1.24 | 1.02 | 1.51 | 0.03 |
| RBP2b | **1.37** | **1.06** | **1.76** | **0.015** | 1.14 | 0.93 | 1.39 | 0.21 |
| RBP2b | **1.42** | **1.10** | **1.83** | **0.006** | 1.25 | 1.02 | 1.53 | **0.032** |
| RBP2cNB | **1.35** | **1.06** | **1.72** | **0.014** | 0.99 | 0.81 | 1.23 | 0.96 |
| RBP2cNB | 1.10 | 0.86 | 1.39 | 0.45 | 1.02 | 0.84 | 1.25 | 0.82 |
| RBP2-P2 | **1.61** | **1.24** | **2.07** | **<0.001** | 1.37 | 1.11 | 1.69 | **0.003** |
| RBP2-P2 | **1.65** | **1.26** | **2.17** | **<0.001** | 1.35 | 1.06 | 1.71 | **0.013** |
| PVX_094350 M | 1.11 | 0.86 | 1.43 | 0.41 | 1.02 | 0.83 | 1.25 | 0.84 |
| PVX_094350 H | **1.33** | **1.05** | **1.67** | **0.016** | 1.01 | 0.84 | 1.21 | 0.94 |
| AKLP2 M | 1.23 | 0.95 | 1.58 | 0.12 | 1.07 | 0.87 | 1.31 | 0.54 |
| AKLP2 H | **1.56** | **1.23** | **1.99** | **<0.001** | 1.10 | 0.90 | 1.35 | 0.34 |
| PVX_087670 M | 1.16 | 0.91 | 1.49 | 0.23 | 0.97 | 0.79 | 1.18 | 0.73 |
| PVX_087670 H | **1.31** | **1.03** | **1.66** | **0.029** | 0.99 | 0.83 | 1.19 | 0.95 |
| RhopH2 M | 1.14 | 0.89 | 1.47 | 0.30 | 1.05 | 0.85 | 1.29 | 0.68 |
| RhopH2 H | **1.37** | **1.08** | **1.74** | **0.009** | 1.06 | 0.87 | 1.29 | 0.55 |
| PVX_122805 M | 1.19 | 0.93 | 1.53 | 0.17 | 1.08 | 0.87 | 1.33 | 0.48 |
| PVX_122805 H | **1.50** | **1.18** | **1.92** | **0.001** | 1.11 | 0.90 | 1.35 | 0.34 |
| CCp5 M | 1.26 | 0.99 | 1.62 | 0.06 | 1.06 | 0.87 | 1.30 | 0.55 |
| CCp5 H | **1.39** | **1.09** | **1.77** | **0.007** | 1.04 | 0.86 | 1.26 | 0.70 |
| PVX_114330 M | 1.11 | 0.86 | 1.43 | 0.42 | 0.95 | 0.77 | 1.17 | 0.63 |
| PVX_114330 H | **1.37** | **1.09** | **1.73** | **0.006** | 1.05 | 0.87 | 1.26 | 0.64 |
| Pv-fam-a/PVX_088820 M | 1.17 | 0.92 | 1.50 | 0.20 | 0.97 | 0.79 | 1.20 | 0.81 |
| Pv-fam-a/PVX_088820 H | **1.36** | **1.06** | **1.73** | **0.014** | 1.14 | 0.93 | 1.39 | 0.21 |
| Pv-fam-a/PVX_092995 M | 1.23 | 0.96 | 1.56 | 0.10 | 0.97 | 0.79 | 1.19 | 0.77 |
| Pv-fam-a/PVX_092995 H | **1.30** | **1.01** | **1.66** | **0.038** | 1.08 | 0.89 | 1.31 | 0.45 |
| PVX_080665 M | **1.30** | **1.02** | **1.67** | **0.035** | 1.10 | 0.90 | 1.36 | 0.35 |
| PVX_080665 H | **1.56** | **1.23** | **1.98** | **<0.001** | 1.18 | 0.96 | 1.45 | 0.11 |
| RAMA M | 1.04 | 0.82 | 1.31 | 0.75 | 0.85 | 0.70 | 1.02 | 0.09 |
| RAMA H | 0.96 | 0.76 | 1.22 | 0.75 | 0.92 | 0.76 | 1.10 | 0.35 |
| SERA M | **1.32** | **1.03** | **1.70** | **0.029** | 1.17 | 0.96 | 1.43 | 0.13 |
| SERA H | **1.44** | **1.12** | **1.84** | **0.004** | 1.13 | 0.93 | 1.38 | 0.23 |
| EBP M | 1.26 | 0.98 | 1.61 | 0.08 | 1.11 | 0.89 | 1.38 | 0.35 |
| EBP H | **1.49** | **1.18** | **1.88** | **0.001** | 1.14 | 0.93 | 1.40 | 0.21 |
