# Supplementary material for: Identification of highly-protective combinations of Plasmodium vivax recombinant proteins for vaccine development
Source: eLife. 2017 Sep 26;6:e28673. doi: 10.7554/eLife.28673 (PMC5655538; doi:10.7554/eLife.28673)
Supplement: Figure 6—source data 1. — AB50 is the estimated antibody levels associated with a 50% probability of protection from clinical malaria. Antibody levels are measured relative to a 1:100 dilution from a pool of plasma from immune Papua New Guinean adults. Estimates of AB50 are also presented as a proportion of the immune plasma pool. The maximum protection is the protection at an antibody level of 0.01. The median of the correlations of that antigen with the other 37 antigens is presented. [file elife-28673-fig6-data1.docx]

**Figure 6 – source data 1: Antibody levels and 50% protection from clinical malaria.** AB50 is the estimated antibody levels associated with a 50% probability of protection from clinical malaria. Antibody levels are measured relative to a 1:100 dilution from a pool of plasma from immune Papua New Guinean adults. Estimates of AB50 are also presented as a proportion of the immune plasma pool. The maximum protection is the protection at an antibody level of 0.01. The median of the correlations of that antigen with the other 37 antigens is presented.

| **Antigen** | **AB_50_** | | **geometric mean antibody level (GMT)** | **proportion**  **> AB_50_** | **maximum protection** | **median correlation** |
| --- | --- | --- | --- | --- | --- | --- |
|  | Antibody levels (arbitrary units) | % of adult pool |  |  |  |  |
| MSP9 N-term | 7.6 x 10^-5^ | 0.8% | 9.2 x 10^-5^ | 45.2% | 51.3% | 0.180 |
| AMA1 | – | – | 4.1 x 10^-4^ | – | 0.0% | 0.329 |
| MSP3a full | 1.8 x 10^-3^ | 18.8% | 1.2 x 10^-3^ | 24.3% | 50.8% | 0.426 |
| MSP3a block1 | 9.3 x 10^-4^ | 9.3% | 7.9 x 10^-4^ | 35.6% | 56.9% | 0.439 |
| MSP3a block2 | 7.3 x 10^-4^ | 7.3% | 5.4 x 10^-4^ | 27.4% | 50.2% | 0.398 |
| MSP3a N-term | 1.5 x 10^-3^ | 14.9% | 1.3 x 10^-3^ | 34.7% | 52.0% | 0.394 |
| MSP3a C-term | 5.2 x 10^-5^ | 0.5% | 1.1 x 10^-4^ | 67.2% | 52.2% | 0.212 |
| DBPII Sal1 | 1.5 x 10^-3^ | 14.9% | 2.4 x 10^-4^ | 3.9% | 93.3% | 0.174 |
| DBPII AH | 1.7 x 10^-3^ | 16.5% | 2.4 x 10^-4^ | 2.7% | 89.7% | 0.181 |
| MSP1 19 | – | – | 4.7 x 10^-4^ | – | 0.0% | -0.011 |
| DBPII C | 5.5 x 10^-3^ | 54.8% | 2.3 x 10^-4^ | 1.1% | 53.9% | 0.089 |
| DBPII P | 1.7 x 10^-3^ | 16.8% | 2.4 x 10^-4^ | 4.8% | 69.2% | 0.091 |
| DBPII O | 5.5 x 10^-3^ | 55.3% | 3.4 x 10^-4^ | 1.9% | 70.7% | 0.127 |
| PVX_094350 | – | – | 1.4 x 10^-3^ | – | 49.6% | 0.360 |
| PVX_087110 | 1.1 x 10^-3^ | 10.6% | 1.3 x 10^-3^ | 48.2% | 51.9% | 0.282 |
| PVX_087670 | 1.13 x 10^-2^ | 113.5% | 1.7 x 10^-3^ | 2.0% | 49.7% | 0.408 |
| PVX_099930 | – | – | 1.4 x 10^-3^ | – | 49.0% | 0.312 |
| PVX_122805-02 | 4.3 x 10^-3^ | 42.7% | 2.0 x 10^-3^ | 11.7% | 50.3% | 0.465 |
| PVX_081330 | 1.3 x 10^-3^ | 13.2% | 1.7 x 10^-3^ | 49.7% | 52.4% | 0.253 |
| PVX_114330 | – | – | 2.1 x 10^-3^ | – | 45.6% | 0.354 |
| PVX_088820 | – | – | 2.3 x 10^-3^ | – | 44.3% | 0.397 |
| PVX_080665 | 5.6 x 10^-3^ | 56.3% | 6.6 x 10^-4^ | 1.3% | 54.5% | 0.369 |
| PVX_092995 | – | – | 1.8 x 10^-3^ | – | 0.0% | 0.434 |
| PVX_087885 | – | – | 1.4 x 10^-3^ | – | 0.0% | 0.264 |
| PVX_003795 | – | – | 9.3 x 10^-4^ | – | 44.9% | 0.326 |
| RBP1a | 4.0 x 10^-4^ | 4.0% | 4.0 x 10^-4^ | 39.0% | 53.2% | 0.242 |
| RBP2a | – | – | 8.5 x 10^-4^ | – | 0.0% | 0.226 |
| RBP2c | – | – | 4.0 x 10^-4^ | – | 0.0% | 0.339 |
| RBP2b | 8.4 x 10^-4^ | 8.4% | 1.2 x 10^-3^ | 55.4% | 66.6% | 0.464 |
| RBP2-P2 | 6.5 x 10^-4^ | 6.5% | 1.7 x 10^-3^ | 73.2% | 54.9% | 0.331 |
| CSP | – | – | 1.4 x 10^-4^ | – | 0.0% | 0.187 |
| ARP | – | – | 4.0 x 10^-4^ | – | 0.0% | 0.253 |
| P41 | 1.8 x 10^-3^ | 17.9% | 1.8 x 10^-5^ | 1.4% | 77.5% | 0.052 |
| PVX_081550 | 3.9 x 10^-5^ | 0.4% | 3.1 x 10^-5^ | 39.1% | 69.3% | 0.099 |
| P12 | – | – | 2.0 x 10^-5^ | – | 0.0% | 0.134 |
| GAMA | – | – | 6.8 x 10^-6^ | – | 0.0% | 0.087 |
| CyRPA | 1.4 x 10^-4^ | 1.37% | 5.2 x 10^-4^ | 60.2% | 69.1% | 0.241 |
| EBP | 3.2 x 10^-4^ | 3.17% | 4.0 x 10^-4^ | 42.7% | 67.3% | 0.210 |
