# Supplementary material for: Identification of highly-protective combinations of Plasmodium vivax recombinant proteins for vaccine development
Source: eLife. 2017 Sep 26;6:e28673. doi: 10.7554/eLife.28673 (PMC5655538; doi:10.7554/eLife.28673)
Supplement: Supplementary file 1. — Conc = concentration; HEK293E = human embryonic kidney 293E cells; WGCF = wheat germ cell-free. *Cd4-tagged proteins. Cd4 alone was conjugated to Luminex beads (2 μg/ml per 2.5 × 10∧6 beads) and tested in all samples as a control for background reactivity. [file elife-28673-supp1.docx]

**Supplementary file 1: *P. vivax* antigens included in this study.** Conc = concentration; HEK293E = human embryonic kidney 293E cells; WGCF = wheat germ cell-free. *Cd4-tagged proteins. Cd4 alone was conjugated to Luminex beads (2 μg/ml per 2.5x10^6 beads) and tested in all samples as a control for background reactivity.

|  | **Antigen** |  | **Region** | **Expression system** | **Reference** | **Conjugation conc/2.5 x 10^6 beads** |
| --- | --- | --- | --- | --- | --- | --- |
| Hypothetical* | StAR-related lipid transfer protein | PVX_081550 | Full ectodomain | HEK293E | (1, 2) | 1.2 μg/mL |
| ARP* | Asparagine-rich protein | PVX_090210 | Full ectodomain | HEK293E | (1, 2) | 0.09 μg/mL |
| GAMA* | GPI-anchored micronemal antigen | PVX_088910 | Full ectodomain | HEK293E | (1, 2) | 0.06 μg/mL |
| P41* | 6-cysteine protein | PVX_000995 | Full ectodomain | HEK293E | (1, 2) | 0.5 μg/mL |
| P12* | 6-cysteine protein | PVX_113775 | Full ectodomain | HEK293E | (1, 2) | 0.2 μg/mL |
| CyRPA | Cysteine-rich protective antigen | PVX_090240 | Full ectodomain | *Baculovirus* |  | 7.5 μg/mL |
| CSP | Circumsporozoite protein | Vaccine construct VMP001 | Chimeric repeated region of VK210 and VK247 variants | *Escherichia coli* | (3, 4) | 1 μg/mL |
| MSP9 | Merozoite surface protein 9 | AF435853 | N-terminus; Amino acids 34–193 | *Escherichia coli* | (5) | 4 μg/mL |
| DBP | Duffy binding protein | P22290.2 | Region II-IV; Variant Sal1 | *Escherichia coli* | (6, 7) | 4 μg/mL |
| DBP |  | AAL79073.1 | Region II; Variant P | *Escherichia coli* | (6, 7) | 4 μg/mL |
| DBP |  | AAL79072.1 | Region II; Variant O | *Escherichia coli* | (6, 7) | 4 μg/mL |
| DBP |  | AAL79060.1 | Region II; Variant C | *Escherichia coli* | (6, 7) | 4 μg/mL |
| DBP |  | AAY34130.1 | Region II; Variant AH | *Escherichia coli* | (6, 7) | 4 μg/mL |
| MSP3α | Merozoite surface protein 3 alpha | AF093584 | Full ectodomain; Nucleotides 1739-4186 | *Escherichia coli* |  | 2 μg/mL |
| MSP3α |  | AF093584 | Block 1; Nucleotides 1982-2908 | *Escherichia coli* |  | 2 μg/mL |
| MSP3α |  | AF093584 | Block 2; Nucleotides 2912-3724 | *Escherichia coli* |  | 2 μg/mL |
| MSP3α |  | AF093584 | C-terminus; Nucleotides 3725-4189 | *Escherichia coli* |  | 2 μg/mL |
| MSP3α |  | AF093584 | N-terminus; Nucleotides 1739-1975 | *Escherichia coli* |  | 2 μg/mL |
| MSP1 | Merozoite surface protein 1 | EDL45115 | MSP1 19, Amino acids L1639-S1729 | *Escherichia coli* |  | 2 μg/mL |
| AMA1 | Apical membrane antigen 1 |  | Full ectodomain | *Escherichia coli* |  | 0.36 μg/mL |
| RBP1a | Reticulocyte binding protein 1a | PVX_098585 | Amino acids 160-1170 | *Escherichia coli* | (8, 9) | 2 μg/mL |
| RBP2a | Reticulocyte binding protein 2a | PVX_121920 | Amino acids 160-1135 | *Escherichia coli* | (8-10) | 17.22 μg/mL |
| RBP2b | Reticulocyte binding protein 2b | PVX_094255 | Amino acids 161-1454 | *Escherichia coli* | (8, 9) | 0.16 μg/mL |
| RBP2cNB | Reticulocyte binding protein 2c without binding domain | PVX_090325 | Amino acids 501-1300 | *Escherichia coli* | (8, 9) | 2 μg/mL |
| RBP2-P2 | Reticulocyte binding protein 2-P2 | PVX_101590 | Amino acids 161-641 | *Escherichia coli* | (8, 9) | 5.4 μg/mL |
| Hypothetical |  | PVX_094350 | Full ectodomain | WGCF | (11) | 3.32 μg/mL |
| AKLP2 | Adenylate  kinase-like protein 2 | PVX_087110 | Full ectodomain | WGCF | (11) | 0.85 μg/mL |
| Hypothetical |  | PVX_087670 | Full ectodomain | WGCF | (11-13) | 1.41 μg/mL |
| RhopH2 | High molecular weight rhoptry protein 2 | PVX_099930 | Full ectodomain | WGCF | (11, 14) | 2.35 μg/mL |
| Hypothetical |  | PVX_122805 | Full ectodomain | WGCF | (11) | 1.79 μg/mL |
| CCp5 | LCCL domain-containing protein | PVX_081330 | Full ectodomain | WGCF | (11) | 1.88 μg/mL |
| Hypothetical | *Plasmodium falciparum* CPW-WPC domain containing protein | PVX_114330 | Full ectodomain | WGCF | (11, 12) | 1.33 μg/mL |
| Pv-fam-a | Tryptophan-rich antigen | PVX_088820 | Full ectodomain | WGCF | (11, 15) | 2.84 μg/mL |
| Pv-fam-a |  | PVX_092995 | Full ectodomain | WGCF | (11, 12) | 1.11 μg/mL |
| Hypothetical |  | PVX_080665 | Full ectodomain | WGCF | (11) | 1.42 μg/mL |
| RAMA | Rhoptry-associated membrane antigen | PVX_087885 | Full ectodomain | WGCF | (11, 12, 16) | 0.2 μg/mL |
| SERA | Serine repeat antigen | PVX_003795 | Full ectodomain | WGCF | (11, 12) | 1.64 μg/mL |
| EBP | Erythrocyte binding protein | KMZ83376.1 | Region II | *Escherichia coli* | (17) | 1.5 μg/mL |

References:

1. Hostetler JB, Sharma S, Bartholdson SJ, Wright GJ, Fairhurst RM, Rayner JC. A Library of Plasmodium vivax Recombinant Merozoite Proteins Reveals New Vaccine Candidates and Protein-Protein Interactions. PLoS Negl Trop Dis. 2015;9(12):e0004264.

2. Franca CT, Hostetler JB, Sharma S, White MT, Lin E, Kiniboro B, et al. An Antibody Screen of a Plasmodium vivax Antigen Library Identifies Novel Merozoite Proteins Associated with Clinical Protection. PLoS Negl Trop Dis. 2016;10(5):e0004639.

3. Bell BA, Wood JF, Bansal R, Ragab H, Cargo J, 3rd, Washington MA, et al. Process development for the production of an E. coli produced clinical grade recombinant malaria vaccine for Plasmodium vivax. Vaccine. 2009;27(9):1448-53.

4. Bennett JW, Yadava A, Tosh D, Sattabongkot J, Komisar J, Ware LA, et al. Phase 1/2a Trial of Plasmodium vivax Malaria Vaccine Candidate VMP001/AS01B in Malaria-Naive Adults: Safety, Immunogenicity, and Efficacy. PLoS Negl Trop Dis. 2016;10(2):e0004423.

5. Lima-Junior JC, Tran TM, Meyer EV, Singh B, De-Simone SG, Santos F, et al. Naturally acquired humoral and cellular immune responses to Plasmodium vivax merozoite surface protein 9 in Northwestern Amazon individuals. Vaccine. 2008;26(51):6645-54.

6. Cole-Tobian JL, Michon P, Dabod E, Mueller I, King CL. Dynamics of asymptomatic Plasmodium vivax infections and Duffy binding protein polymorphisms in relation to parasitemia levels in Papua New Guinean children. Am J Trop Med Hyg. 2007;77(5):955-62.

7. Cole-Tobian JL, Michon P, Biasor M, Richards JS, Beeson JG, Mueller I, et al. Strain-specific duffy binding protein antibodies correlate with protection against infection with homologous compared to heterologous plasmodium vivax strains in Papua New Guinean children. Infect Immun. 2009;77(9):4009-17.

8. Hietanen J, Chim-Ong A, Chiramanewong T, Gruszczyk J, Roobsoong W, Tham WH, et al. Gene Models, Expression Repertoire, and Immune Response of Plasmodium vivax Reticulocyte Binding Proteins. Infect Immun. 2015;84(3):677-85.

9. Franca CT, He WQ, Gruszczyk J, Lim NT, Lin E, Kiniboro B, et al. Plasmodium vivax Reticulocyte Binding Proteins Are Key Targets of Naturally Acquired Immunity in Young Papua New Guinean Children. PLoS Negl Trop Dis. 2016;10(9):e0005014.

10. Gruszczyk J, Lim NT, Arnott A, He WQ, Nguitragool W, Roobsoong W, et al. Structurally conserved erythrocyte-binding domain in Plasmodium provides a versatile scaffold for alternate receptor engagement. Proc Natl Acad Sci U S A. 2016;113(2):E191-200.

11. Arumugam TU, Ito D, Takashima E, Tachibana M, Ishino T, Torii M, et al. Application of wheat germ cell-free protein expression system for novel malaria vaccine candidate discovery. Expert Rev Vaccines. 2014;13(1):75-85.

12. Lu F, Li J, Wang B, Cheng Y, Kong DH, Cui L, et al. Profiling the humoral immune responses to Plasmodium vivax infection and identification of candidate immunogenic rhoptry-associated membrane antigen (RAMA). J Proteomics. 2014;102:66-82.

13. Chen JH, Chen SB, Wang Y, Ju C, Zhang T, Xu B, et al. An immunomics approach for the analysis of natural antibody responses to Plasmodium vivax infection. Mol Biosyst. 2015;11(8):2354-63.

14. Wang B, Lu F, Cheng Y, Li J, Ito D, Sattabongkot J, et al. Identification and characterization of the Plasmodium falciparum RhopH2 ortholog in Plasmodium vivax. Parasitol Res. 2013;112(2):585-93.

15. Wang B, Lu F, Cheng Y, Chen JH, Jeon HY, Ha KS, et al. Immunoprofiling of the tryptophan-rich antigen family in Plasmodium vivax. Infect Immun. 2015;83(8):3083-95.

16. Changrob S, Wang B, Han JH, Lee SK, Nyunt MH, Lim CS, et al. Naturally-Acquired Immune Response against Plasmodium vivax Rhoptry-Associated Membrane Antigen. PLoS One. 2016;11(2):e0148723.

17. Ranjan A, Chitnis CE. Mapping regions containing binding residues within functional domains of Plasmodium vivax and Plasmodium knowlesi erythrocyte-binding proteins. Proc Natl Acad Sci U S A. 1999;96(24):14067-72.
