# Supplementary material for: Identification of highly-protective combinations of Plasmodium vivax recombinant proteins for vaccine development
Source: eLife. 2017 Sep 26;6:e28673. doi: 10.7554/eLife.28673 (PMC5655538; doi:10.7554/eLife.28673)
Supplement: Transparent reporting form [file elife-28673-transrepform.docx]

***eLife’s* transparent reporting form**

We encourage authors to provide detailed information *within their submission* to facilitate the interpretation and replication of experiments. If you have any questions, please contact us: [editorial@elifesciences.org](mailto:editorial@elifesciences.org).

**Sample-size estimation**

- You should state whether an appropriate sample size was computed when the study was being designed
- You should state the statistical method of sample size computation and any required assumptions
- If no explicit power analysis was used, you should describe how you decided what sample (replicate) size (number) to use

Please outline where this information can be found within the submission (e.g., page numbers or figure legends), or explain why this information doesn’t apply to your submission:

Power analysis was not used in the present study. The present study made use of samples collected as part of a longitudinal cohort study of 264 children conducted in Papua New Guinea in 2006 (Lin et al. PLOS one 2010), and all available samples (N = 225) of children who completed the 16 months of follow-up were tested.

Detailed information on the cohort study and the samples tested in the present study can be found in the “Study participants and ethical approval” section of Material and Methods, page 22.

**Replicates**

- You should report how often each experiment was performed
- You should include a definition of biological versus technical replication
- The data obtained should be provided and sufficient information should be provided to indicate the number of independent biological and/or technical replicates
- If you encountered any outliers, you should describe how these were handled
- Criteria for exclusion/inclusion of data should be clearly stated
- High-throughput sequence data should be uploaded before submission, with a private link for reviewers provided (these are available from both GEO and ArrayExpress)

Please outline where this information can be found within the submission (e.g., page numbers or figure legends), or explain why this information doesn’t apply to your submission:

Detailed information on validation, assay and controls used can be found in the “Antibody measurement” and “Statistical analysis” sections of Material and Methods, page 24.

**Statistical reporting**

- Statistical analysis methods should be described and justified
- Raw data should be presented in figures whenever informative to do so (typically when N per group is less than 10)
- For each experiment, you should identify the statistical tests used, exact values of N, definitions of center, methods of multiple test correction, and dispersion and precision measures (e.g., mean, median, SD, SEM, confidence intervals; and, for the major substantive results, a measure of effect size (e.g., Pearson's r, Cohen's d)
- Report exact p-values wherever possible alongside the summary statistics and 95% confidence intervals. These should be reported for all key questions and not only when the p-value is less than 0.05.

Data processing and statistical analysis applied can be found in the sections “Statistical analysis”, “Simulated annealing for investigating combinations of antigens”, “Dose-response relationship” of Material and Methods, pages 24-26. Statistical tests/measure of effect size used, numbers, dispersion and precision measures, confidence intervals, and P values are described throughout Results, pages 8-14; as well as within the figures, tables and/or their legends.

Please outline where this information can be found within the submission (e.g., page numbers or figure legends), or explain why this information doesn’t apply to your submission:

(For large datasets, or papers with a very large number of statistical tests, you may upload a single table file with tests, Ns, etc., with reference to page numbers in the manuscript.)

**Additional data files (“source data”)**

- We encourage you to upload relevant additional data files, such as numerical data that are represented as a graph in a figure, or as a summary table
- Where provided, these should be in the most useful format, and they can be uploaded as “Source data” files linked to a main figure or table
- Include model definition files including the full list of parameters used
- Include code used for data analysis (e.g., R, MatLab)
- Avoid stating that data files are “available upon request”

Please indicate the figures or tables for which source data files have been provided:

The covariates and parameters included in our statistical and mathematical models are described in the “Statistical analysis”, “Simulated annealing for investigating combinations of antigens” and “Dose-response relationship” sections of Material and Methods, page 24.

In order to present as much source data as possible, we included the following: details on the proteins used have been included in Supplementary file 1, in complement to the described in the “Antigen selection” section of Material and Methods (page 22); complementary data for Figure 1 has been provided in Table 1; detailed/complementary data for Figure 2 has been provided in Figure 2-source data 1; data for Figure 3 has been detailed in Figure 3-source data 1, and further complemented in Figure 3-source data 2; complementary data for figure 6 has been provided in Figure 6-figure supplement 1 and Figure 6-source data 1.

The full database used is available upon reasonable request by contacting the

PNG Medical Research Advisory Committee and the PNG Institute of Medical Research IRB, in order to avoid compromise participant privacy and violate the ethical agreement in the informed consent forms. The contact is Dr. William Pomat, secretary PNG IMR IRB: William.Pomat@pngimr.org.pg.
